# Excellent Rectifying Properties of the n-3C-SiC/p-Si Heterojunction Subjected to High Temperature Annealing for Electronics, MEMS, and LED Applications

**DOI:** 10.1038/s41598-017-17985-9

**Published:** 2017-12-18

**Authors:** Philip Tanner, Alan Iacopi, Hoang-Phuong Phan, Sima Dimitrijev, Leonie Hold, Kien Chaik, Glenn Walker, Dzung Viet Dao, Nam-Trung Nguyen

**Affiliations:** 1 Queensland Micro and Nanotechnology Centre, Griffith University, Queensland, 4111 Australia; 2 School of Engineering, Griffith University, Queensland, 4111 Australia

## Abstract

This work examines the stability of epitaxial 3C-SiC/Si heterojunctions subjected to heat treatments between 1000 °C and 1300 °C. Because of the potential for silicon carbide in high temperature and harsh environment applications, and the economic advantages of growing the 3C-SiC polytype on large diameter silicon wafers, its stability after high temperature processing is an important consideration. Yet recently, this has been thrown into question by claims that the heterojunction suffers catastrophic degradation at temperatures above 1000 °C. Here we present results showing that the heterojunction maintains excellent diode characteristics following heat treatment up to 1100 °C and while some changes were observed between 1100 °C and 1300 °C, diodes maintained their rectifying characteristics, enabling compatibility with a large range of device fabrication. The parameters of as-grown diodes were J_0_ = 1 × 10^−11^ A/mm^2^, n = 1.02, and +/−2V rectification ratio of 9 × 10^6^. Capacitance and thermal current-voltage analysis was used to characterize the excess current leakage mechanism. The change in diode characteristics depends on diode area, with larger areas (1 mm^2^) having reduced rectification ratio while smaller areas (0.04 mm^2^) maintained excellent characteristics of J_0_ = 2 × 10^−10^ A/mm^2^, n = 1.28, and +/−2V ratio of 3 × 10^6^. This points to localized defect regions degrading after heat treatment rather than a fundamental issue of the heterojunction.

## Introduction

The unique enabling capabilities of silicon carbide due to its superior electrical and physical properties make it attractive for new generations of commercially viable products. SiC wafers of the hexagonal polytypes, 4 H and 6 H with their wide band gaps of 3.23 eV and 3.05 eV respectively, and high voltage breakdown characteristics are being fabricated into Schottky diodes and MOSFETs for power conversion and conditioning, providing improved efficiency and reduced system size and weight. Commercialization of these devices occurred following the improvement in bulk crystal quality and epilayers and in the case of MOSFETs, the development of nitrided gate oxides to achieve better yields, performance and reliability^1,2^. Despite the extremely high SiC wafer cost and small wafer size that make these devices expensive, products are now commercially available and their market is growing due to the ever increasing global drive to reduce energy consumption and harmful emissions.

Similarly, the advancement in technology for the deposition of silicon carbide on silicon has also been evolving, with the 3C polytype being most commonly investigated because device quality films can be grown between 1000 °C and 1380 °C, below the silicon melt temperature^3,4^. This polytype can be deposited on silicon wafers in various forms including epitaxial, polycrystalline, nanocrystalline through to amorphous^5–7^. The ability to deposit on large silicon wafers that enables the use of advanced and mature low cost silicon fabrication technologies to fabricate a wide range of devices makes for a compelling argument for this material to be adopted as a new platform technology in a host of applications. Its superior attributes can provide a commercial advantage to a wide range of better and lower cost competitive and enabling products. The use of 3C-SiC on Si for products such as MEMs and bio-mems^8^, ultra-thin membranes for e-beam and x-ray applications^9^, discrete devices such as pressure^10^, piezoresistive^11–13^ and temperature sensors^14^, photovoltaics^15^ and photonics^16,17^ are all possible and can provide significant product benefits. LED’s may also benefit from SiC on Si technology, as the lattice constant match of SiC with AlN and GaN provides epitaxial deposition compatibility. Thus, 3C-SiC on (111)Si can be used as a template for the hexagonal nitride growth^18^. The change from the cubic SiC structure to hexagonal structure of the nitrides can inhibit crystal defect propagation through the interface leading to a reduction in crystal defects in the GaN and AlGaN layers. For this application, conduction through the heterojunction is required and can be facilitated by an n-SiC/n-Si junction. The (100) cubic SiC can be used as a template for the cubic growth of GaN, with its non-polar nature being reported to provide a path for further improvements in green LED performance^19^.

Yet even the best efforts to produce high quality epitaxial films have not been able to completely eliminate certain crystal defects and film stress, due to the inherent problems of lattice mismatch (20%) and different thermal expansion coefficients (20% at 1200 °C) between SiC and Si. Crystal defects, most commonly stacking faults near the interface have been reduced over time and optimization of the initial cleaning, carbonization, and growth steps has resulted in significant improvements in overall film quality^3,20^. Despite the presence of stacking faults, they should not pose a problem electrically. The cubic form of SiC has a band gap of 2.36 eV which is probably the lowest gap of any of the 200 or so SiC polytypes. Stacking faults would represent higher band gap material and therefore the material band gap is determined by the 3C polytype. There are a number of contradictory papers that discuss the effectiveness of SiC as a Si and C diffusion barrier. The formation of Si voids at the SiC/Si interface is well documented and its occurrence is cited as indirect proof of Si diffusion into the SiC during deposition^3,20^. Some publications suggest that diffusion of Si and C even in polycrystalline SiC where the grain boundaries provide preferential diffusion paths should be almost non-existent below 1400 °C^21^. However, once an effective barrier film is formed we have seen no evidence of subsequent void formation. The deposition quality and application specific customization of the process as well as improvements in deposition reactors will be required to meet the exacting requirements of product development and its use in production.

Despite the presence of crystal defects at the interface, the n-SiC/p-Si heterojunction has been shown to exhibit high quality diode properties^22–25^, with the large valence band energy difference between the Si and SiC forming a very effective barrier to holes flowing from the p-Si into the SiC which makes it useful in heterojunction bipolar transistors^26^. For many other applications, electrical isolation of the SiC from the Si substrate can be exploited by the use of the n-SiC/p-Si heterojunction. There has recently been some controversy in the literature regarding the stability of this heterojunction at higher temperatures^27,28^. It was claimed that the carbonization barrier can break down at temperatures roughly above 1000–1100 °C, and that this effect is so prominent that the rectifying n-SiC/p-Si junction is destroyed and the heterojunction becomes electrically shorted^27^. Thus, it is claimed, the interface instability has crucial consequences for applications exposed to high temperatures such as SiC on Si for harsh environments, or its use as a substrate for growth of III-N compounds and graphene. The implication of this casts doubt over the physical and electrical stability and robustness of the 3C-SiC/Si interface when exposed to temperatures above 1000 °C. While the operational temperature in service is likely to be several hundreds of degrees below 1000 °C, these higher temperatures may well occur in some later fabrication steps.

This led us to a systematic investigation into the effect of thermal treatments on the SiC/Si heterojunction in which we developed a sensitive test structure that could be exposed to high temperatures and thus determine the onset of changes at the heterojunction. Any change may also indicate if silicon or carbon diffusion in either the Si or SiC was occurring. While optical examination can be used to reveal the presence of voiding at the SiC/Si interface, a much more sensitive detection method is via electrical testing in the form of current-voltage and capacitance-voltage measurements. In this study we aim to establish at what temperature we begin to see degradation of the SiC/Si heterojunction and whether it remains of sufficiently high quality and robust at high temperatures to be useful in the above applications.

## 3C-SiC Epilayers and Electrical Test Structures

**Figure 1:**
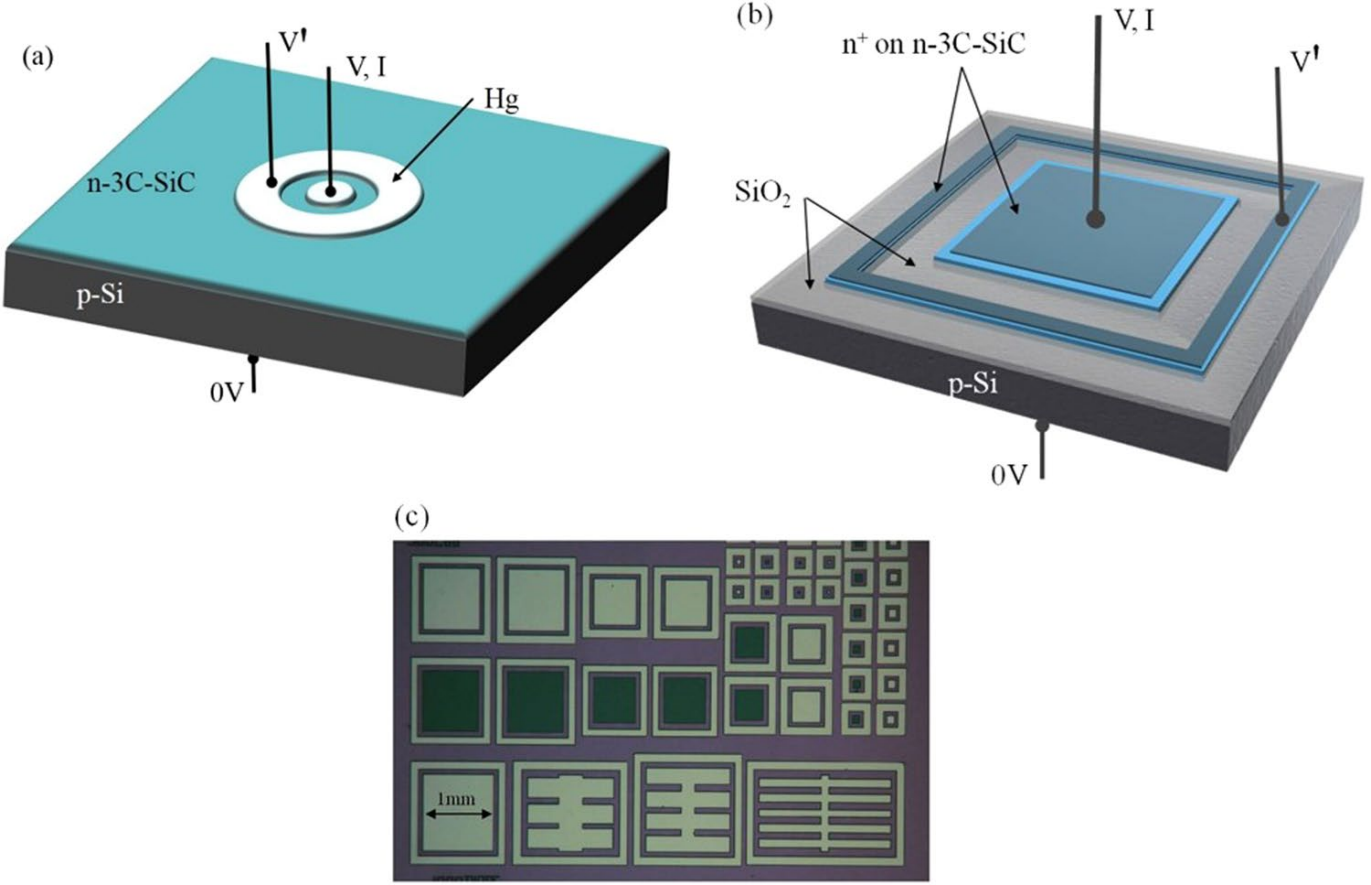
Test structures showing (**a**) Hg probe configuration with central dot and guard ring, (**b**) plasma etched mesa and guard ring with SiO_2_ sidewall passivation, and (**c**) plan view of the mesa test structures with different sizes and shapes. The largest square measures 1 × 1 mm.

As part of an experimental matrix, a number of 150 mm wafers had been processed with a variety of thermal conditions whereby approximately 400 nm of 3C-SiC, unintentionally n-doped via residual nitrogen in the system, was deposited on the front and back surfaces of p-Si wafers using an SPT Epiflx SiC epitaxial LPCVD reactor designed for large batch production runs of up to 50 wafers with a diameter of 300 mm. In samples described as blanket films, all carbonization and initial SiC deposition was performed at or below 1000 °C, followed by further bulk SiC deposition at either 1000 °C or 1200 °C. A third blanket film underwent bulk deposition at 1200 °C and then was annealed at 1300 °C for 3 hours before unloading from the reactor. This group of samples was then analyzed electrically using an MDC mercury probe and Keithley 6430 picoammeter after removing SiC from the back surface and sputtering Al as a back contact. The mercury probe had a central dot electrode of area 0.48 mm^2^ and a surrounding guard ring, as shown in Fig. 1(a). With the back of the sample grounded, current-voltage data (V,I) was measured at the dot, while the ring electrode voltage(V′) followed the dot voltage as a means to eliminate lateral surface currents.

In other samples, SiC films grown at 1000 °C were plasma etched to form isolated SiC areas followed by passivation of the sidewalls and surrounding surfaces with silicon oxide and further deposition of a highly doped n-SiC top electrode that was suitable for probing and also capable of being annealed at very high temperature without reacting with the underlying low doped SiC and affecting the SiC/Si interface (Fig. 1(b)). In order to accurately measure the expected sub-nanoamp heterojunction currents, it is important to eliminate perimeter leakage of these mesas, or otherwise be able to separate areal and perimeter leakage through measurement. This was achieved by patterning diodes of different sizes, or different shapes (same area, different perimeter lengths) as shown in Fig. 1(c) and incorporating guard ring structures to reduce lateral surface leakage. Optimisation of the processes by oxide passivation of the Si led to significant improvement in the reverse bias leakage where no perimeter or surface leakage effects were seen. As a final process step, the back contact was formed on the Si substrate with sputtered Al annealed in a rapid thermal processor at 500 °C for 5 minutes.

Mesa structures were measured with a Keysight B1505A parameter analyser connected to an enclosed, temperature controlled probe station. The assessment of the heterojunction was performed by analysis of the forward bias (SiC more negative than Si) and reverse bias (SiC more positive than Si) currents of mesas measured before and after annealing. The same meter was used to measure capacitance-frequency responses at 0 V bias.

## Results and Discussion

**Figure 2:**
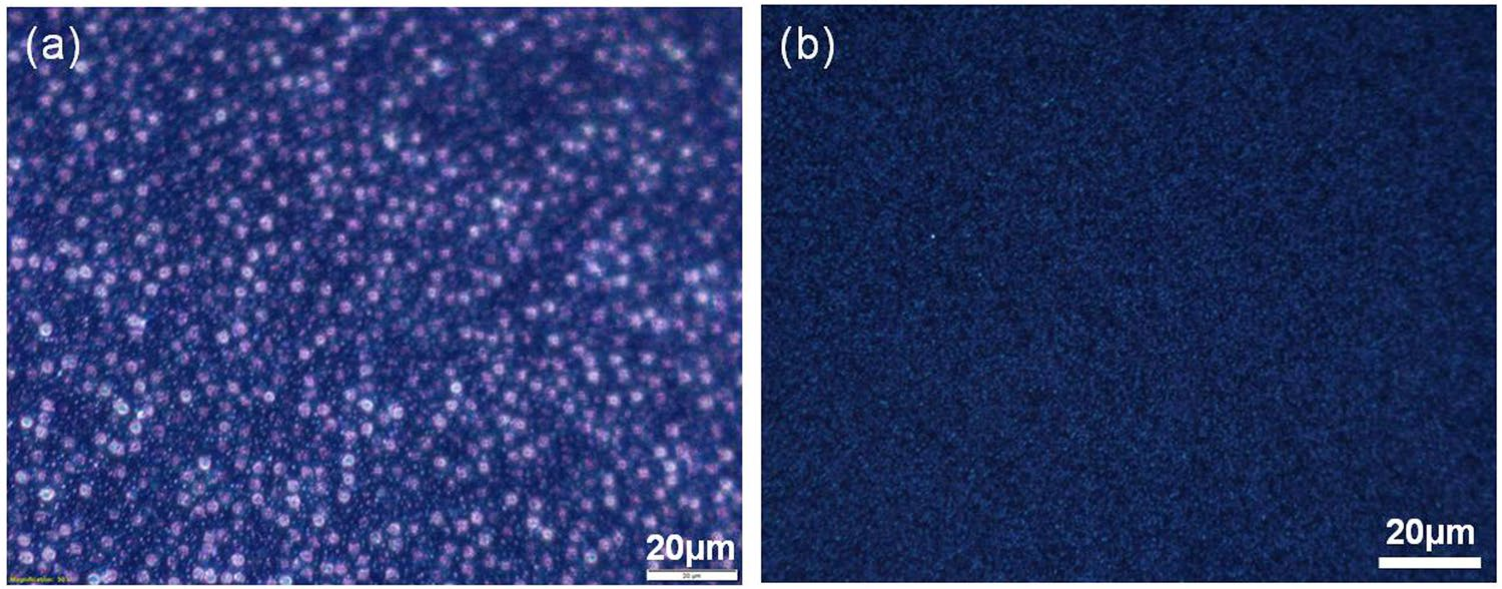
Dark field optical microscope images of (**a**) 3C-SiC non-ideal growth with a high density of interface voids and (**b**) optimised growth followed by 1250 °C anneal for 2 hours.

Variation in the epitaxial SiC deposition process can change the crystal quality, doping and surface roughness of the SiC film. For a film grown in non-optimized conditions, a commonly observed effect is voiding in the Si immediately under the SiC film, as seen in Fig. 2(a). To examine the physical integrity of the interface, voiding was assessed for each annealed sample by optical microscope under dark field and observed to be completely black with 100x magnification. Typically in 3C-SiC epitaxial films grown on Si, voiding is seen at high densities with a uniform distribution of triangles (111) or squares (100) dependent upon the Si crystal orientation. With high illumination and using a high sensitivity CCD camera with long exposures (5 s) by focusing on defects the dark field image is shown to be unaffected by annealing even up to 1250 °C (Fig. 2(b)). The texture seen with these long exposures is thought to be slight surface roughness.

**Figure 3:**
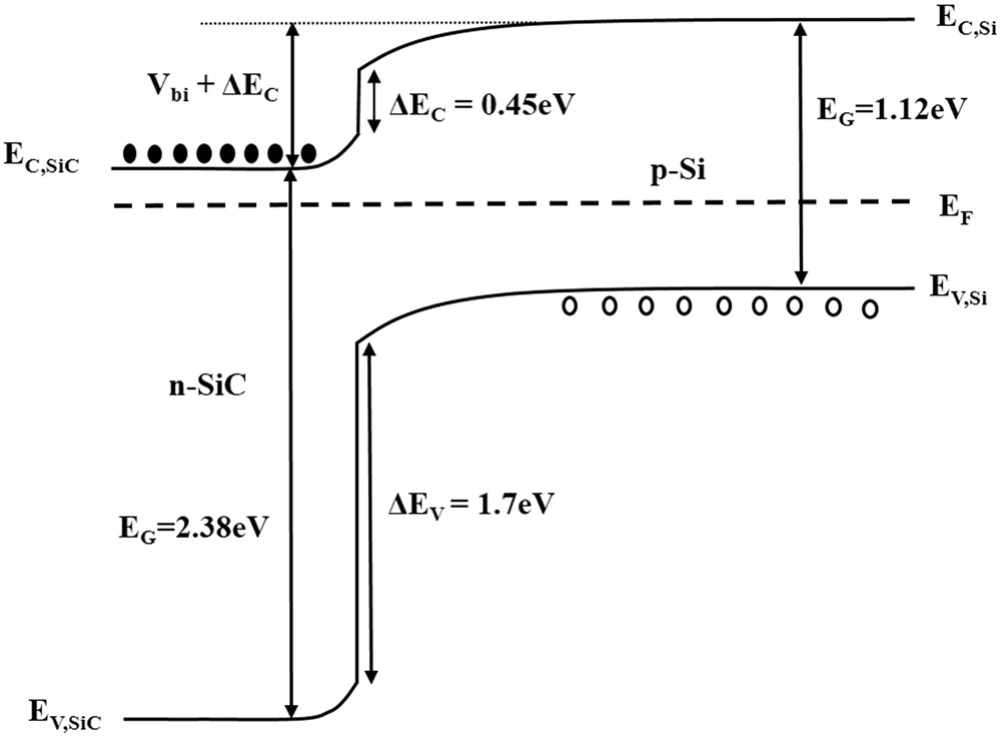
Energy band diagram of the n-3C-SiC/p-Si heterostructure at 0 V bias. The proposed current mechanisms under forward and reverse bias are depicted in Supplementary Fig. S1.

One of the most sensitive methods of detecting changes at the SiC/Si interface is via electrical measurements. Current-voltage characteristics of the heterojunction can be compared with the expected ideal result and modelled according to well established theories governing processes such as thermal emission, drift/diffusion, generation/recombination, and tunneling. Figure 3 depicts the energy band diagram for the n-3C-SiC/p-Si heterojunction based on the findings of Afanas’ev and co-workers^29^ who found ΔE_C_ of 0.45 eV and ΔE_V_ of 1.7 eV using an internal photoemission technique. A number of studies have been conducted on this type of heterostructure and propose various current mechanisms including trap assisted tunneling^30^, multistep recombination-tunneling^31,32^, and tunneling-thermal emission^22^. These various mechanisms are shown in Supplementary Fig. S1 for the forward and reverse bias cases.

**Figure 4:**
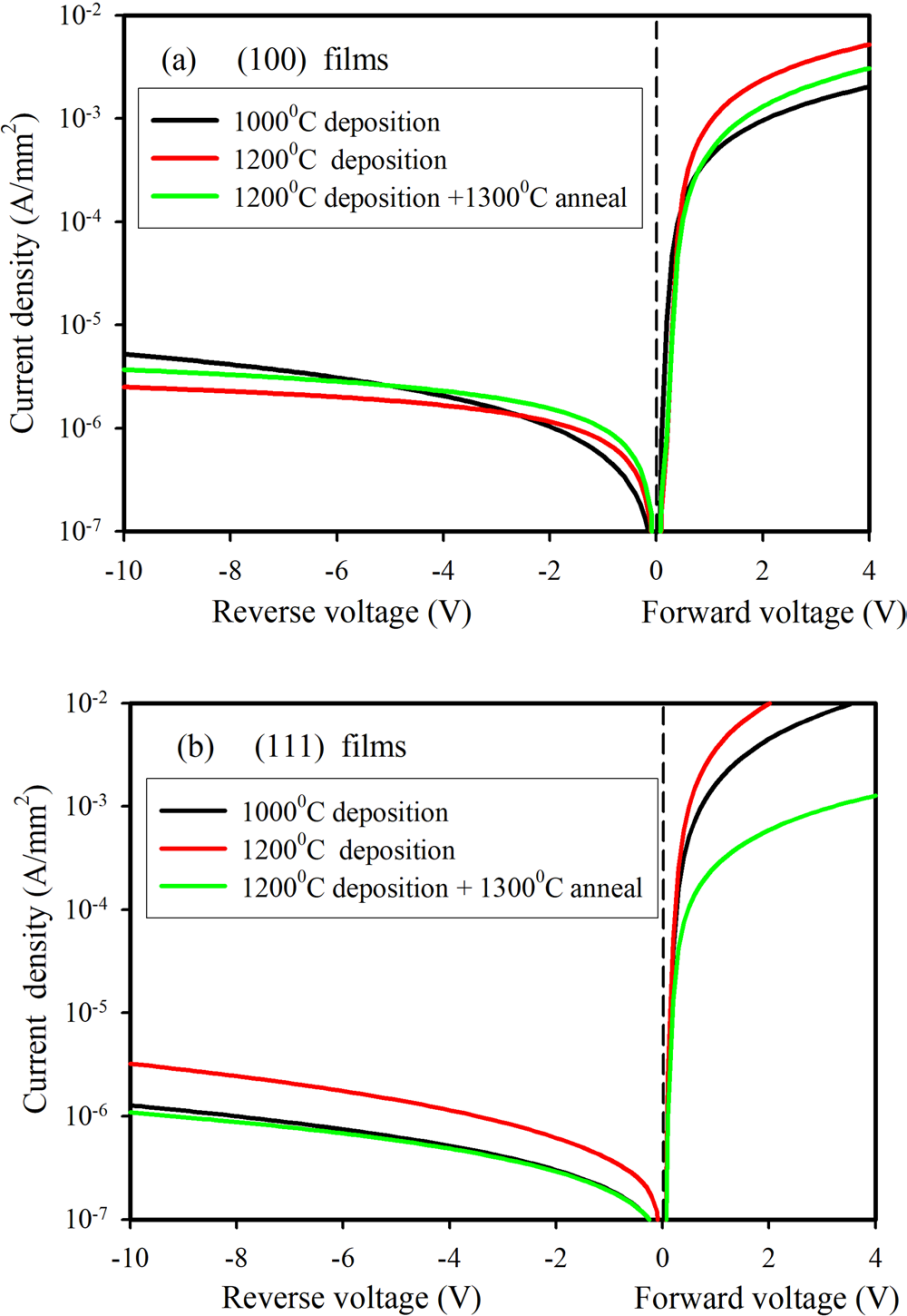
Current-voltage characteristics of 3C-SiC blanket films deposited on (**a**) (100)Si and (**b**) (111) Si at 1000 °C and 1200 °C, and deposited at 1200 °C and then annealed at 1300 °C. Samples were measure by mercury probe with dot and guard ring configuration. Dot area is 0.48 mm^2^.

Current-voltage measurements using a mercury probe of blanket SiC films grown on (100) and (111) oriented Si substrates are shown in Fig. 4. It can be seen that all samples exhibited some degree of rectifying behaviour, even a film deposited at 1200 °C and annealed at 1300 °C for 3 hours. In the case of (100) films, +/−2 V rectification ratios were in the range 840–2000 while for (111) films values of 2020–16000 were measured. There was no obvious trend with temperature that we attributed to the different deposition and anneal conditions. These results are in direct contradiction to other reports claiming that the heterojunction can be catastrophically destroyed by heat treatments above 1100 °C^27^.

**Figure 5:**
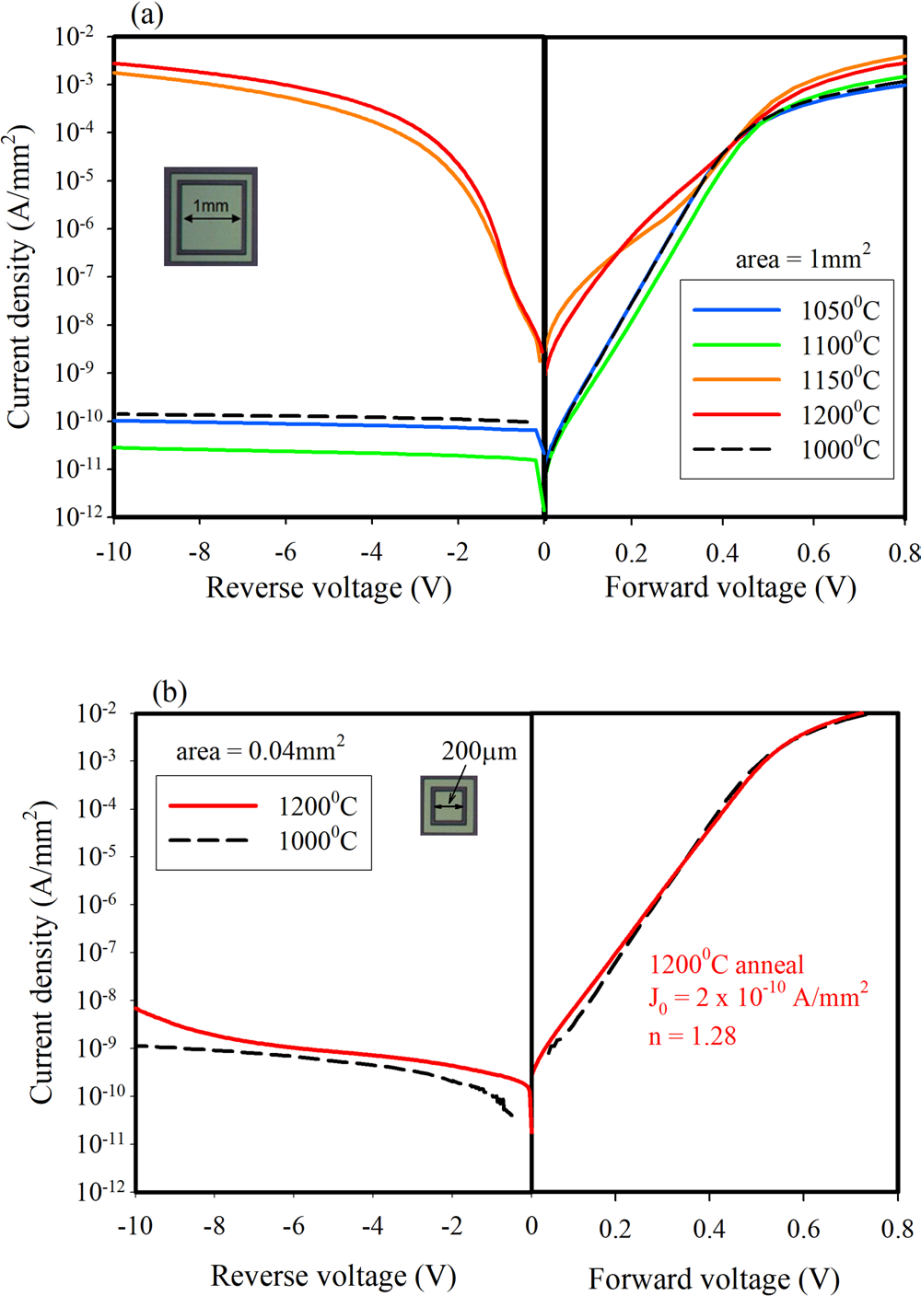
(**a**) Forward and reverse bias Log J-V plots of 1 mm^2^ diodes submitted to five different anneal temperatures from 1000 °C to 1200 °C, and (**b**) plots of smaller area devices (0.04 mm^2^) annealed at 1000 °C and 1200 °C showing very little degradation of the heterojunction.

In order to perform a more rigorous study of the heterojunction, the structures of Fig. 1(b) and (c) were fabricated using (100) oriented films, whereby defined areas or mesas were created and the sidewalls passivated by silicon oxide. SiC films grown at 1000 °C were further processed at 1000 °C for 2 hours during the growth of a highly doped n-type SiC electrode layer. Different fragments of the wafer were then annealed at 1050 °C, 1100 °C, 1150 °C, and 1200 °C for two hours in nitrogen atmosphere and electrical measurements performed as described above. The forward bias current density-voltage characteristics can be modelled using the basic diode equation^23^, modified to include the effect of series resistance,

1 $$J={J}_{0}[exp(\frac{q(V-J{R}_{S})}{nkT})-1]$$

where

2 $${J}_{0}\propto exp(-\frac{{E}_{a}}{kT})$$

where J_0_ is the reverse saturation current density, n the diode ideality factor, k is Boltzmann’s constant, and R_S_ the series resistance of the diode and its contacts. In the absence of traps at the interface, the value of J_0_ is determined by the thermal emission of electrons over the energy barrier V_bi_ + ΔE_C_ (see Fig. 3) followed by recombination with holes in the p-Si, and the value of n will be 1. The presence of traps acting as recombination centres in the space charge region will increase the value of J_0_ and also increase n towards a value of 2. The thermal emission of holes from the valence band of the Si to that of the SiC can be ignored due to the much larger energy barrier. The diode currents have a certain temperature dependence or activation energy, E_a_, that gives a good indication of the presence of traps/defects at the interface that influence trap assisted recombination and hence increase the leakage current and reduce the activation energy. As shown in Fig. 5(a), the heterojunctions maintain excellent diode properties for anneal temperatures up to 1100 °C with J_0_ and n in the range 1–3 × 10^−11^ A/mm^2^ and 1.00–1.08 respectively, while those annealed at 1150 °C and 1200 °C begin to show degradation as evidenced by excess current leakage in reverse bias and low forward bias. Nevertheless, the rectification ratio at +/−2V as shown in Table 1 for each anneal temperature still indicates a minor physical degradation of the interface rather than any fundamental gross change or intermixing of the two materials.

**Table 1:** The current rectification ratio at +/−2V for each anneal temperature of 1 mm^2^ diodes, and the case of a 0.04 mm^2^ diode annealed at 1200 °C.

Anneal temperature (°C)	Diode area (mm^2^)	+/−2V rectification ratio
1000	1.00	9.0 × 10^6^
1050	1.00	8.9 × 10^6^
1100	1.00	1.4 × 10^7^
1150	1.00	1.3 × 10^3^
1200	1.00	7.2 × 10^2^
1200	0.04	3.0 × 10^6^

The current leakage uniformity across the sample was investigated by comparing square devices with areas from 1 mm^2^ down to 0.04 mm^2^ for the 1200 °C anneal sample. The larger areas showed J-V plots similar to the 1200 °C anneal case in Fig. 5(a), while a number of smaller devices (0.04 mm^2^) were found to have degraded only slightly after the anneal with J_0_ = 2 × 10^−10^ A/mm^2^, n = 1.28, and +/−2V ratios of 3 × 10^6^ as seen in Fig. 5(b). A device with the same area that was annealed at 1000 °C is shown for comparison. This indicates that the areas of degraded SiC/Si interface are not uniformly distributed on a micron sized scale, but rather that areas of thousands of square micrometers remain very stable after high temperature processing.

The increased reverse bias leakage in the case of the 1150 °C and 1200 °C annealed diodes was investigated to determine how much the edge of the device was contributing to the leakage current. For the 1200 °C anneal case, devices with the same area (1 mm^2^) and increasing perimeter lengths (4 mm to 20 mm) were measured and seen to have a very weak dependence of reverse bias leakage on perimeter length. A fivefold increase in perimeter length resulted in only a 15% increase in leakage current, indicating that the body of the diode is responsible for the majority of the excess leakage.

**Figure 6:**
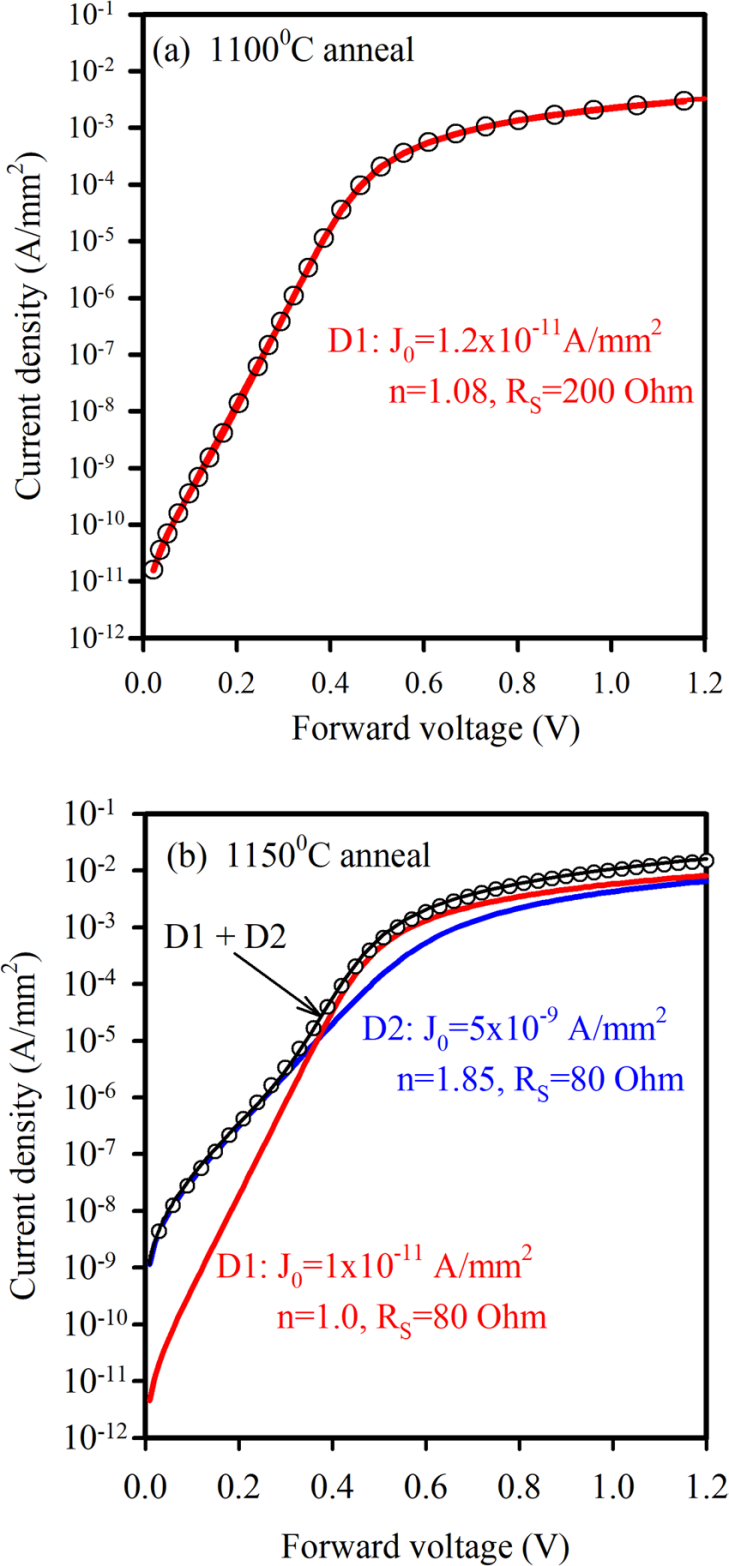
Measured(dots) and modelled(lines) J-V plots of 1 mm^2^ diodes annealed at (**a**) 1100 °C and (**b**) 1150 °C. The sample annealed at 1100 °C can be modelled by a single diode, while the sample annealed at 1150 °C is modelled by two parallel diodes with different J_0_ and n values.

While an ideal junction will have low J_0_ and n = 1 due to pure thermal emission of electrons over the conduction band energy barrier (see supplementary Fig. S1(a)), non-ideal junctions have n > 1 or non-linear forward biased Log J-V plots and increased reverse bias currents. Based on the results presented in Fig. 5, the post-anneal increase in low forward bias and reverse bias currents is not uniformly distributed across the junction area. Thus we propose that only a portion of the diode area has degraded, and so the J-V plot can be modelled by two or more sets of parameters. This degradation can be due to the presence of electrically active defects (traps) acting as generation-recombination centres in the space charge region surrounding the junction, thus causing the increased trap-assisted currents (see Supplementary Fig. S1(a) and (b)). As an example, Fig. 6 compares the forward bias J-V plots for 1 mm^2^ diodes annealed at 1100 °C and 1150 °C. In the former case, Fig. 6(a), a single diode is adequate to model the device while in the latter case, Fig. 6(b), two parallel diodes provide an excellent fit to the measured data. The first diode (D1) has very similar parameters to the 1100 °C anneal case, while the second diode (D2) has a J_0_ value 500 times higher and n = 1.85 indicating an increase in the recombination current mechanism. The R_S_ values are quite high, due to the non-optimised back contact, but they are still at a reasonable level to allow determination of J_0_ and n.

**Figure 7:**
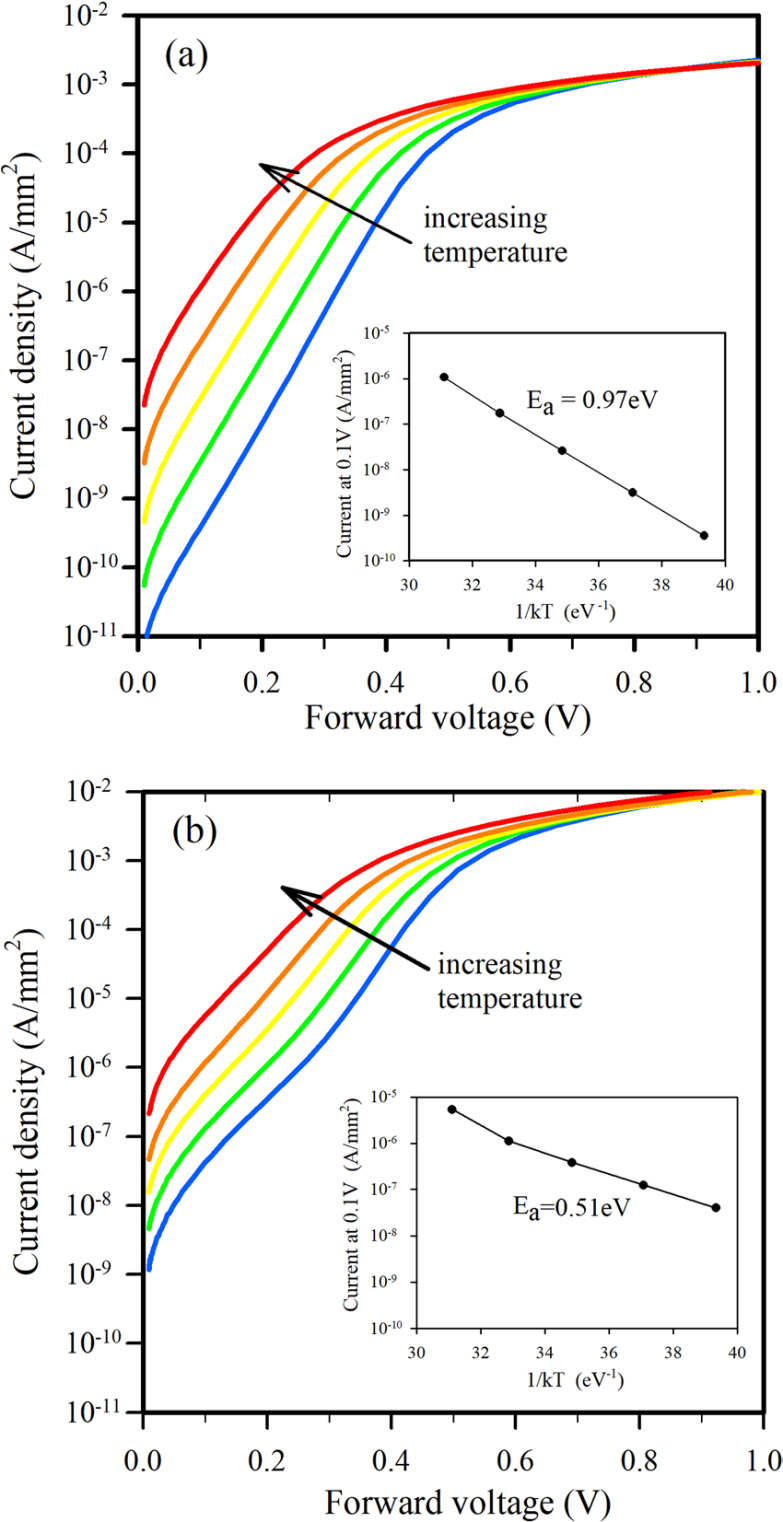
Forward J-V plots measured at 22 °C, 40 °C, 60 °C, 80 °C, and 100 °C for 1 mm^2^ devices annealed at (**a**) 1100 °C, and (**b**) 1150 °C. Inserts show Arrhenius plots and the activation energies of currents at 0.1 V.

The temperature dependence of the forward bias currents can be used to find the energy location of defects responsible for the added leakage currents. In the 1100 °C anneal case, Fig. 7(a) shows the current-voltage plots at various temperatures with the inset showing that the activation energy of the current at 0.1 V is 0.97 eV, defined as the slope of the Arrhenius plot (Ln J versus 1/kT). This indicates that the dominant mechanism is thermal emission of electrons over the energy barrier V_bi_ + ΔE_C_ of the junction as depicted in Supplementary Fig. S1(a). After the 1150 °C anneal, currents at 0.1 V displayed an activation energy of 0.51 eV (Fig. 7(b)), close to the Si midgap. One would expect defects to exhibit a range of energies through the Si energy gap, with those near the midgap having the highest probability of acting as recombination centres. In the reverse bias case at 1 V, a similar trend was seen with the activation energies being 0.96 eV and 0.43 eV for the 1100 °C and 1150 °C anneals respectively. The higher activation energy is close to the Si bandgap energy (1.12 eV) indicating that minority carrier generation in the p-Si dominates the reverse current while the lower activation energy is likely due to the trap assisted tunnelling-thermal emission processes depicted in Supplementary Fig. S1(b).

To confirm the dominant current mechanism in reverse bias after high temperature annealing (1150–1200 °C), the J-V data of Fig. 5(a) was analyzed in terms of the tunneling or field emission equation^33^,

3 $${I}_{R}=C{V}_{R}^{\kappa }exp(-\frac{B}{{V}_{R}})$$

where C, B, and κ are constants. Equation 3 can be rearranged to give,

4 $$-\frac{dLn({I}_{R})}{d(\frac{1}{{V}_{R}})}=\kappa {V}_{R}+B$$

**Figure 8:**
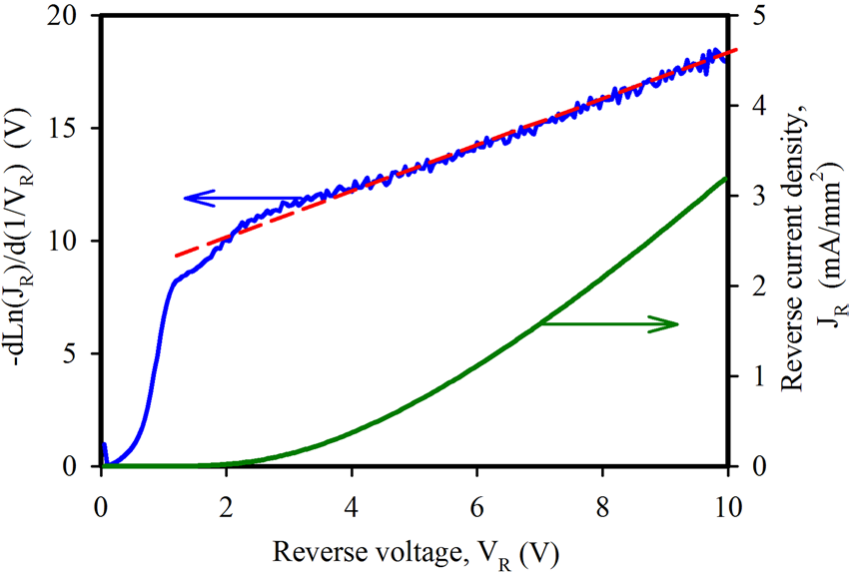
Data from the 1200 °C annealed sample shown in Fig. 5(a) plotted according to the tunneling or field emission equation of Eq. 4. The linear fit (dashed line) for V_R_ > 2 V indicates that tunneling is dominant.

Thus, a plot of $-\frac{dLn({I}_{R})}{d(1/{V}_{R})}$ versus V_R_ will be linear if tunneling is the dominant mechanism. Figure 8 shows the data of a 1 mm^2^ diode that was annealed at 1200 °C plotted in this way, and it is evident that for voltages greater than 2 V there is indeed a fairly close fit to the linear relationship of Eq. 4, as shown by the dashed line. This result indicates that tunneling is the dominant reverse current mechanism in this case.

**Figure 9:**
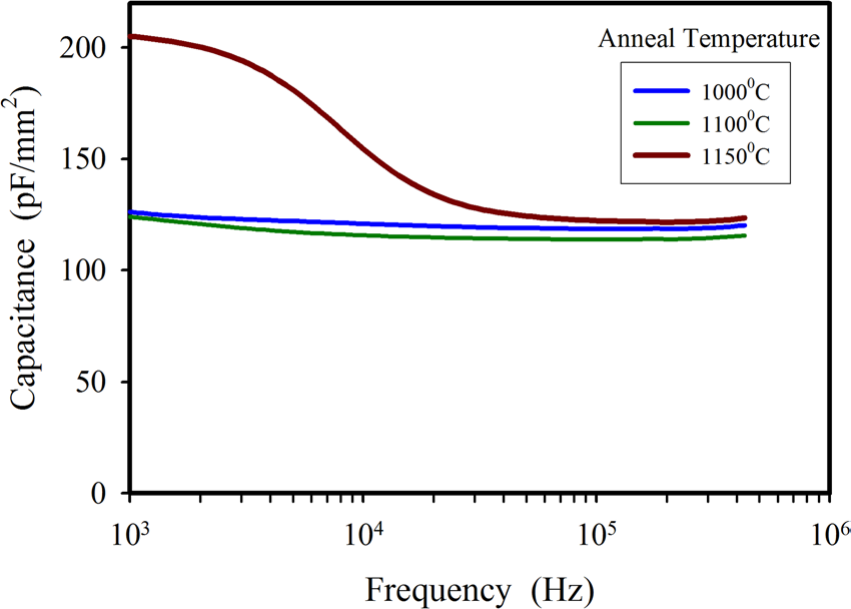
Capacitance-frequency response of devices annealed at 1000 °C, 1100 °C, and 1150 °C.

Capacitance measurements are often used to detect the presence of electrically active defects in semiconductor structures. In the ideal SiC/Si heterojunction, the measured capacitance is determined by the depletion width which exists mainly in the lower doped Si side of the junction. The presence of any interface traps will add to the value of this capacitance at frequencies where they are fast enough to respond to the AC signal. Figure 9 compares the 0 V bias capacitance-frequency plots of devices annealed at 1000 °C, 1100 °C, and 1150 °C. For the two lower temperature anneals, the C-f response is almost constant indicating very few interface traps, whereas the 1150 °C anneal has increased capacitance at lower frequencies due to the presence of traps.

In conclusion, we have investigated the stability of the n-3C-SiC/p-Si heterojunction as it is subjected to anneal temperatures from 1000 °C to 1300 °C in a nitrogen atmosphere. Initially, blanket films grown on either (100) or (111) oriented Si were annealed at temperatures up to 1300 °C and found to maintain a good degree of rectification in the current-voltage curve. The +/−2V rectification ratios were 840 and 2020 respectively for the (100) and (111) films after a 1300 °C anneal, a finding that contradicts the claim that temperatures above 1100 °C can cause catastrophic failure of the heterojunction. Optical microscope images revealed an absence of interface voids that are a common problem when 3C-SiC is grown on Si.

Different (100) films were then studied more closely by forming isolated mesa structures with oxide passivated sidewalls and a heavily doped 3C-SiC top electrode layer. Diodes with 1 mm^2^ area showed excellent J-V characteristics after annealing at temperatures up to 1100 °C, with sub-nanoamp reverse bias currents and +/−2V rectification ratios in the 10^6^–10^7^ range. Above 1100 °C, the J-V characteristics of larger area devices (1 mm^2^) began to show non-ideal behaviour as evidenced by a double diode type characteristic in the forward bias region and increased reverse bias leakage. By measuring diodes of different areas and shapes, it was determined that the excess leakage current was not edge related and the J-V relationship indicated an enhanced tunnelling process in the body of the device at reverse voltages above 2 V. Despite this observed change in the electrical behaviour of the junction, and without optimizing the ohmic contacts of the device, a +/−2V rectification ratio of 720 was measured after a 1200 °C anneal of 1 mm^2^ mesa structures. Importantly, some smaller devices (0.04 mm^2^) maintained their excellent diode characteristics after a 1200 °C anneal, with J_0_ = 2 × 10^−10^ A/mm^2^, n = 1.28, and +/−2V ratios of 3 × 10^6^. This dependence of diode parameters on device area indicates the presence of localised degradation of the interface rather than a fundamental issue of the heterojunction. Capacitance-voltage measurements confirmed the interface changes after anneal temperatures greater than 1100 °C, with increased capacitance at lower frequencies due to the presence of interface traps. Future investigations will focus on determining the nature and cause of these localised defects.

This sensitive electrical test verifies that even a 3C-SiC/Si heterojunction that has not been optimized for this purpose can survive high temperature annealing above 1000 °C thereby making 3C-SiC on Si useful in a range of applications where the junction may be subject to extreme temperatures as part of the fabrication requirement such as the processing of III-V materials and graphene growth.

## Methods

### 3C-SiC Deposition

The 3C-SiC epitaxial growth began with 150 mm silicon wafers being loaded from the nitrogen load lock into the SiC reactor at 500 °C before being evacuated to the 10^−7^ mbar range. The temperature was then ramped to 1000 °C while flowing 1.2 L/minute of oxygen that maintained the thin clean oxide on the silicon surface. Silane was then introduced at 4sccm ensuring a partial pressure regime where SiO_2_ was etched from the Si surface, followed by epitaxial silicon growth on silicon. To ensure a void free silicon under the SiC film, the activated silicon surface was then carbonised at 950 °C by a 10sccm C_3_H_6_ flow rate and the temperature ramped back to 1000 °C. An Alternate Supply Epitaxy (ASE) growth cycling method^4^ was then used to grow uniform (<+/−1%) high quality 3C-SiC by flowing 7sccm SiH_4_ for 70 s followed by a flow of 40 sccm C_3_H_6_ for 20 s at the specified growth temperature. This procedure was then repeated until the desired thickness of SiC had been achieved. Films of approximately 400 nm thickness were deposited with n-type doping concentration of 10^16^–10^17^cm^−3^ on p-Si with doping concentration of 5 × 10^14^ cm^−3^.

### Mesa Fabrication

In order to fabricate the mesa test structures, the 3C-SiC was patterned using conventional lithography processes before plasma etching the SiC to create mesas using HCl and/or SF_6_ in a STS ICP or a LAM 480 system. The plasma etching proceeded through the SiC and into the Si to ensure that the mesas/islands of SiC were isolated from each other. Following the SiC etch, the photoresist was removed by an oxygen plasma in a Tegal 915 etcher. Piranha cleaning (4:1 H_2_SO_4_:H_2_O_2_ at 90–100 °C for 15 mins.) was used to remove any further residues or contamination from the Si surface before HF etching (1%HF for 2 mins.) to prepare the Si surface for oxide deposition. A Hitech LPCVD furnace was used to deposit a conformal 100 nm Low Temperature Oxide by flowing 20 sccm SiH_4_ and 20 sccm 0_2_ at a pressure of 100 mT and a temperature of 440 °C for 5 mins. Windows were then opened in the oxide to expose more than 90% of the SiC mesa top using a buffered oxide etch. The resist was again stripped off using the Tegal system and any further residues removed using piranha etch. By using ammonia as the precursor, 100 nm or 300 nm of highly doped n-SiC was then selectivity deposited on the exposed areas of the SiC mesa using a Hitech LPCVD reactor at 1000 °C to provide a high conductivity area for probing. The wafer was then cleaved into die of 30mmx30mm so that a thermal anneal matrix could be carried out on material from the same wafer.

### Sample Annealing

Annealing was carried out in a Hitech® LPCVD furnace with a ramp rate of 2°/min from 1000 °C to the anneal temperature with a 100 sccm flow of nitrogen. The temperature was then held at the anneal temperature for 2 hours before cooling at 3°/min.

### Data Availability

The datasets generated during and/or analysed during the current study are available from the corresponding author on reasonable request.

## Electronic supplementary material

Figure S1
