# Supplementary material for: Excellent Rectifying Properties of the n-3C-SiC/p-Si Heterojunction Subjected to High Temperature Annealing for Electronics, MEMS, and LED Applications
Source: Sci Rep. 2017 Dec 18;7:17734. doi: 10.1038/s41598-017-17985-9 (PMC5735178; doi:10.1038/s41598-017-17985-9)
Supplement: Supplementary file 1 — Figure S1 [file 41598_2017_17985_MOESM1_ESM.pdf]

Excellent Rectifying Properties of the n-3C-SiC/p-Si Heterojunction Subjected to High Temperature Annealing for Electronics, MEMS, and LED Applications

Philip Tanner, Alan Iacopi, Hoang-Phuong Phan, Sima Dimitrijev , Leonie Hold, Kien Chaik , Glenn Walker, Dzung Viet Dao , Nam-Trung Nguyen.

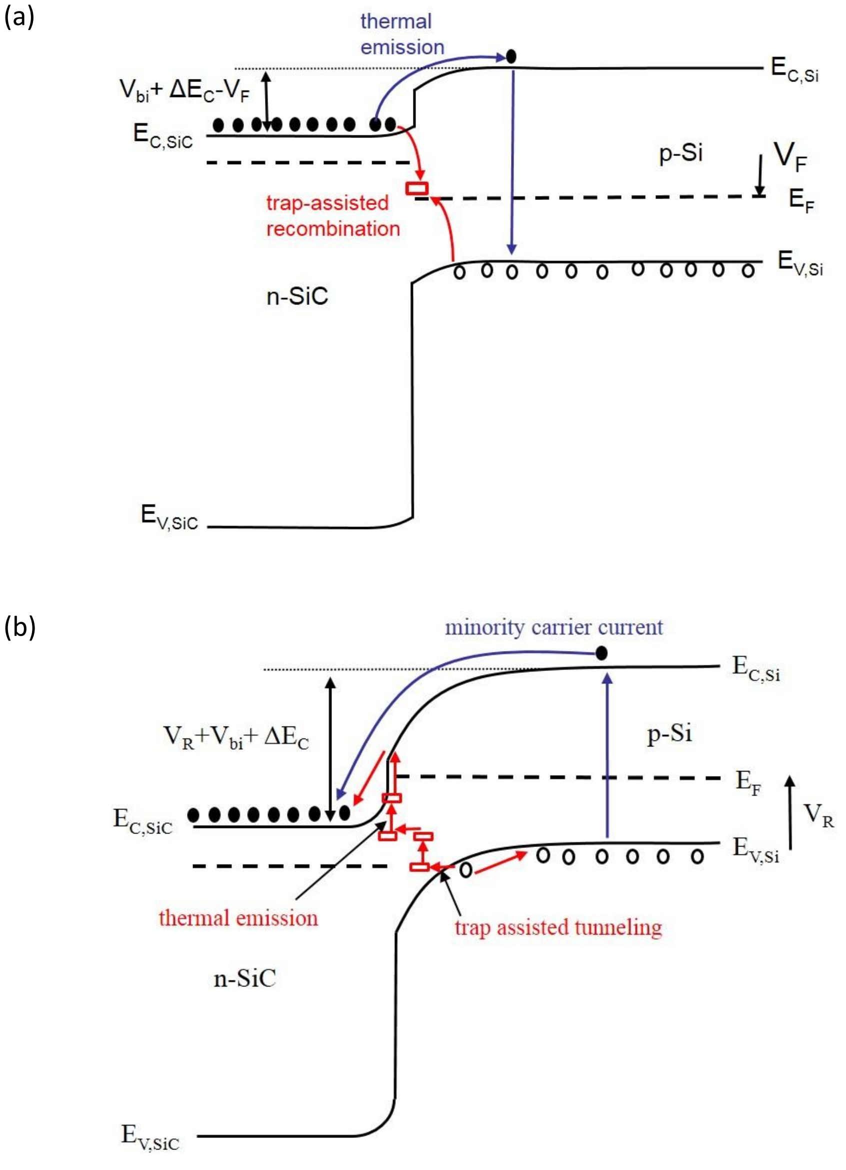

Fig. S1. Energy band diagrams of the n-3C-SiC/p-Si heterojunction showing the main current mechanisms. (a) In forward bias, V_F, thermal emission of electrons over the energy barrier in the conduction band followed by recombination with holes in the p-Si (blue), and trap assisted recombination in the space charge region (red). (b) In reverse bias, V_R, minority carrier current via thermal generation of electrons in the p-Si followed by drift/diffusion into the SiC (blue), and trap assisted tunneling/thermal emission (red).
